# Highly photoluminescent and stable silicon nanocrystals functionalized *via* microwave-assisted hydrosilylation†

**DOI:** 10.1039/c7ra13577g

**Published:** 2018-03-12

**Authors:** Deski Beri, Dmitry Busko, Andrey Mazilkin, Ian A. Howard, Bryce S. Richards, Andrey Turshatov

**Affiliations:** Institute of Microstructure Technology, Karlsruhe Institute of Technology Hermann-von-Helmholtz-Platz 1, 76344, Eggenstein-Leopoldshafen Germany andrey.turshatov@kit.edu bryce.richards@kit.edu; Institute of Nanotechnology, Karlsruhe Institute of Technology Hermann-von-Helmholtz-Platz 1, 76344 Eggenstein-Leopoldshafen Germany; Light Technology Institute, Karlsruhe Institute of Technology Engesserstrasse 13 76131 Karlsruhe Germany; Chemistry Department, Universitas Negeri Padang Jl. Hamka Air Tawar 25131 Indonesia

## Abstract

Herein, we report a microwave-assisted hydrosilylation (MWH) reaction for the surface passivation of silicon nanocrystals (Si-NCs) with linear alkenes. The MWH reaction requires only 20 minutes and allows us to produce Si-NCs with high photoluminescence quantum yields (PLQYs), reaching 39% with an emission maximum of 860 nm. Furthermore, we investigated the effect of ligand length on the photoluminescence properties of Si-NCs. We tested six alkenes with an even number of carbon atoms (from hexene-1 to hexadecene-1). The highest PLQY combined with a long stability (test period of 6 months) was observed when capping with the shortest ligand, hexene-1. The use of microwave heating turns the hydrosilylation step into a facile and sustainable process. In order to provide insight into the emissive properties of Si-NCs surface oxidation and luminescence decay were investigated using Fourier-transform infrared spectroscopy and time-resolved photoluminescence measurements.

## Abstract 2

Herein, we report a microwave-assisted hydrosilylation (MWH) reaction for the surface passivation of silicon nanocrystals (Si-NCs) with linear alkenes.

## Graphical abstract

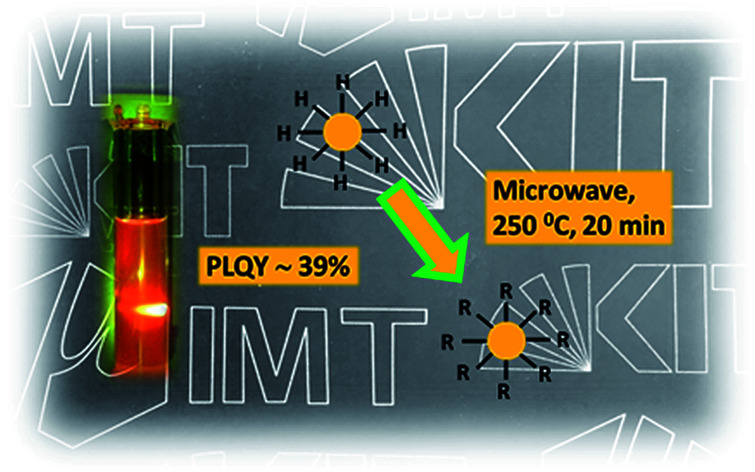

## Introduction

Silicon nanocrystals (Si-NCs) have attracted much research interest over the last few decades because of their potential applications in optoelectronics, photovoltaics, and photonics, as well as in the biomedical field for cancer treatment.^1^ Silicon (Si) is an extremely abundant and non-toxic element contained within the earth's crust.^2^ Bulk crystalline silicon (c-Si) exhibits an indirect band gap; in order to emit a photon a conduction-band electron needs phonon assistance to recombine with a valence-band hole. This means that photon emission in c-Si is slow, hence non-radiative recombination processes usually dominate and, as such, c-Si is a poor light emitter. However, when c-Si becomes finely divided onto a nanometer scale close to its free exciton Bohr radius (4.3 nm for bulk c-Si), its photophysics are altered due to the quantum confinement that takes place. In this regime, the electron no longer needs a phonon to recombine with the hole, so Si-NC are able to emit light in the red or near-infrared (NIR) region. Nevertheless, due to increased reactivity of Si-NCs towards oxygen and water molecules, the synthesis of Si-NCs with high photoluminescence quantum yield (PLQY) is challenging.

There are two the promising methods to produce large quantities of luminescent Si-NCs, namely synthesis in a non-thermal plasma reactor or high temperature disproportionation reaction of silicon oxide based materials – silicon monoxide SiO_*x*_ or hydrogen silsesquioxanes (HSiO_1.5_)_*n*_ – and subsequent functionalization of the hydrogen capped nanocrystals (H–Si-NCs) with organic ligands.^3–12^ The functionalization of Si-NCs relies on radical hydrosilylation reactions, initiated by temperature, light or radical initiators. The functionalization is necessary as it significantly increases the PLQY and chemical stability of the Si-NCs. The aforementioned method is able to provide suspensions of Si-NCs with maximum PLQY of 20–35% with emission in the NIR region.^13–16^

There are several reports with extraordinarily high PLQYs. For instance, Meinardi *et al.* reported PLQY of 50% for NIR emission of Si-NCs synthesized in a non-thermal plasma reactor and capped with dodecene-1.^17^ A long shelf-life of dodecyl-Si-NCs with PLQY of 62% (the PLQY remained unchanged after two months under ambient conditions) was reported recently by Islam *et al.*^18^ Sangghaleh *et al.* reported PLQY of 60% for Si-NCs (capped with methyl undecanoate) prepared from silsesquioxanes.^19^ Qian *et al.* described that the stability of Si-NCs can be increased by using of fluorinated capping ligands.^20^ In addition, embedding Si-NCs into polymer matrix is very promising way to achieve exceptionally high PLQY (65%) and long-term chemical stability.^21^ Even though high values of PLQY have been reported recently, but still, there is a lack of systematic study of the long-term stability of Si-NCs (shelf-life under ambient conditions).

In our work, we annealed silicon monoxide SiO_*x*_ in reducing atmosphere and elaborated the hydrogen terminated Si-NCs *via* reaction with hydrofluoric acid. We optimized hydroxylation step realized using microwave-assisted hydrosilylation (MWH) and addressed the investigation of photoluminescence and stability of Si-NCs obtained *via* the MWH step. MWH is an advanced process of the Si-NCs functionalisation which is able to reduce reaction time, improving the product quality, product reproducibility and a cleaner synthesis.^4,22^ In our report we demonstrate that at higher temperatures, MWH can run 10–30 times faster than conventional heating and the reaction can yield Si-NCs with high PLQY (up to 35%) and excellent shelf-life (6 months). In addition, we investigated the effect of using alkenes with different length (*N*-ene-1, where *N* is number of carbon atoms in the linear alkyl chain) on the photophysical properties of Si-NCs as well as the shelf-life stability of the product.

## Results and discussion

In order to elucidate the effect of the organic ligands on the luminescent properties of the Si-NCs all chemical steps preceding the hydrosilylation reaction were performed under similar conditions. Reduction of SiO_*x*_ in continuous flow of Ar/H_2_ gas and subsequent etching with 48% HF solution leads to the formation of hydrogen-capped Si-NCs. Subsequently, within 20 minutes these are converted in a microwave reactor to form stable Si-NCs capped with organic ligands. We measured the PLQY of the freshly prepared samples. Afterwards, each sample was divided into two vials. One vial was placed in a glovebox with inert atmosphere and used as a control experiment. The second part of the suspension was stored in the dark in closed vials. The vials were opened regularly for PLQY measurements. Thus, no special precautions were attempt for the storage of the Si-NCs placed in the second vial.

**Fig. 1:**
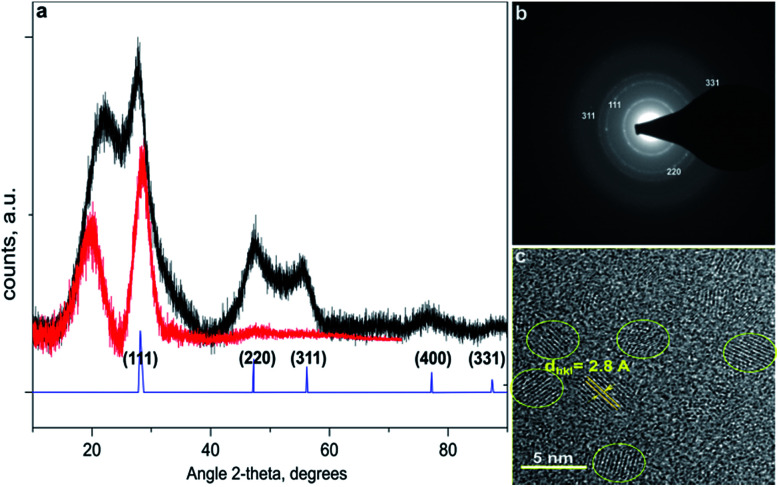
(a) Representative X-ray diffractograms of Si-NCs in SiO_*x*_ matrix before HF etching (black line) and free standing Si-NCs capped with decene-1 along with JCPDS 027-1402; (b) electron diffraction data for free standing Si-NCs capped with decene-1; (c) HRTEM image of free standing Si-NCs capped with decene-1.

**Fig. 2:**
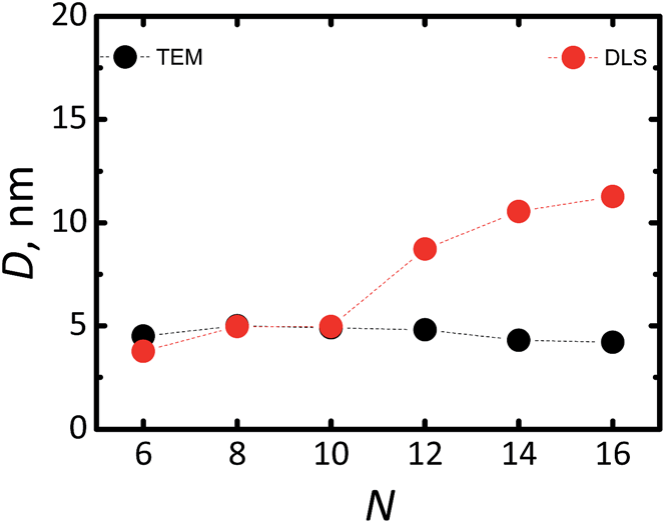
Average diameter of Si-NCs estimated with TEM (black cycles) and DLS (red cycles) as function of number (*N*) of carbon atoms in the linear aliphatic chain of the capping ligand.

The data of Fig. 1a and b reveals diamond cubic crystal structure of synthesized Si-NCs. High resolution transmission electron microscopy (HRTEM) (Fig. 1c) confirms the crystal structure of Si-NCs with interplanar distance of 0.32 nm corresponding to the distance (111) in c-Si. The average size of Si-NCs realized in this work is ∼4.6 ± 0.3 nm (sum of six independent etching processes), as deduced from transmission electron microscopy (TEM) images (see Fig. S1†). Importantly, TEM results indicate that the average size of the Si-NCs core remains the same after the surface passivation step with all investigated ligands (Fig. 2 and S1†). In contrast, the results of DLS measurements (Fig. 2) indicate an increase of the hydrodynamic diameter in case of surface passivation with dodecene-1 and longer hydrocarbons. Previously, Yang *et al.*^23^ reported the existence of a polymer corona forming around the Si-NCs core during the hydrosilylation process. They found that oxygen, high ligand concentration, and increase of temperature promote chain propagation in oligomerization reactions and lead to formation of an organic corona. In our experiment, we observed that the size and the hydrodynamic diameter of the nanocrystals are consistent in case of capping with relatively short ligands, *e.g.* hexene-1, octene-1 and decene-1, whereas the use of the long ligands (with *N* ≥ 12) leads to of the growth of a thick organic shell surrounding the silicon core and a discrepancy between the particle size measured by TEM (seeing only the Si-NCs core) and DLS (seeing the Si-NCs core plus organic corona).

**Fig. 3:**
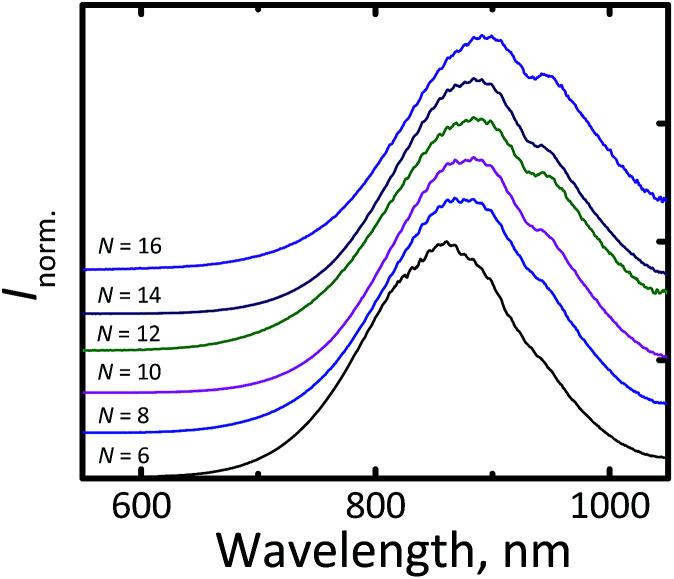
Normalized fluorescent spectra of Si-NCs with different capping ligands. *N* is the number of carbon atoms in the linear aliphatic chain of the capping ligand.

Fig. 3 displays the photoluminescence (PL) spectra of the synthesized Si-NCs. Under ultraviolet (UV, 375 nm) excitation all nanocrystals demonstrate strong emission in the NIR part of the electromagnetic spectrum. The effective mass approximation (EMA)^24,25^ model predicts (eqn (1)^26^) that uniform Si-NCs with size 4.6 nm emit photons with energy *E*_PL_ = 1.55 eV (*λ*_max_ = 800 nm).1
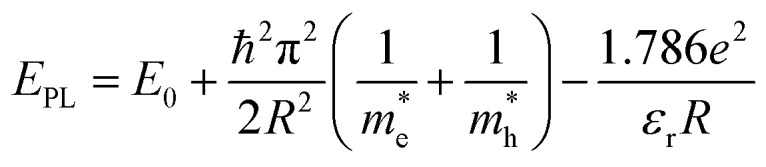
where *E*_0_ is band gap of c-Si, ℏ is Planck constant with stroke, *R* – radius of Si-NCs, 
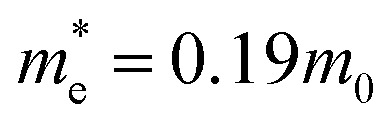
 and 
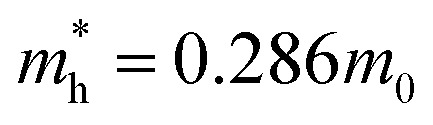
 are effective masses of electrons and holes in Si-NCs correspondingly, *m*_0_ – electron mass, *e* – charge of electron, *ε*_r_ – relative permittivity of Si-NCs.

In our measurements, we observed an unusually large red shift in the position of the luminescence maximum (*λ*_max_ = 850–905 nm) for all ligands, as shown in Fig. 3 (it should be noted that the local minimum at 920 nm can be explained by absorption of the solvent associated with the overtone of C–H stretching vibration). This effect cannot be explained by a special condition (MW heating) of the hydrosilylation process.

The same shift in the position of the emission maximum was observed for Si-NCs prepared by hydrosilylation *via* conventional heating in agreement with experimental procedure described by Sun *et al.*^6^ (see Fig. S2†). We assume that the shift can be explained by a polydispersity of the Si-NCs ensembles. A broad sized distribution results the red shift in a position of an emission maximum in comparison with the theoretical prediction for monodisperse Si-NCs, as it has been recently described by Yu *et al.*^15^ Fig. S3† supports this statement: we observed the clear correlation between FWHM of the emission peak and position of the luminescence maximum. The broader emission peaks is the more red shifted peaks are observed.

**Fig. 4:**
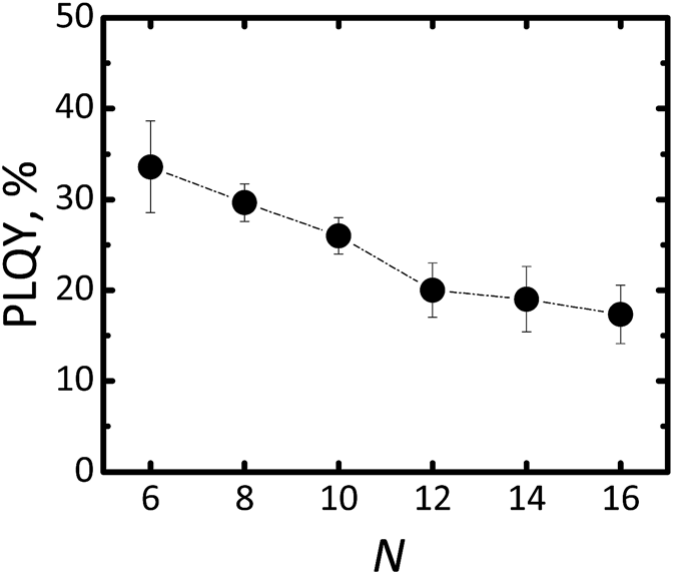
Changing of PLQY for Si-NCs functionalised with different capping ligands. *N* is the number of carbon atoms in the linear aliphatic chain of the capping ligand.

In order to quantify luminescence of Si-NCs we measured absolute PLQY for all samples. The results of the PLQY measurements are summarized in Fig. 4.

The highest PLQY value of 34 ± 4% was observed for capping with the shortest ligand – hexene-1. Importantly, the error bar of PLQY values was derived from three independent experiments for each capping ligand, except for hexene-1 for which six independent experiments were performed. The general trend indicates that the longer the ligand is the lower the PLQY.

**Fig. 5:**
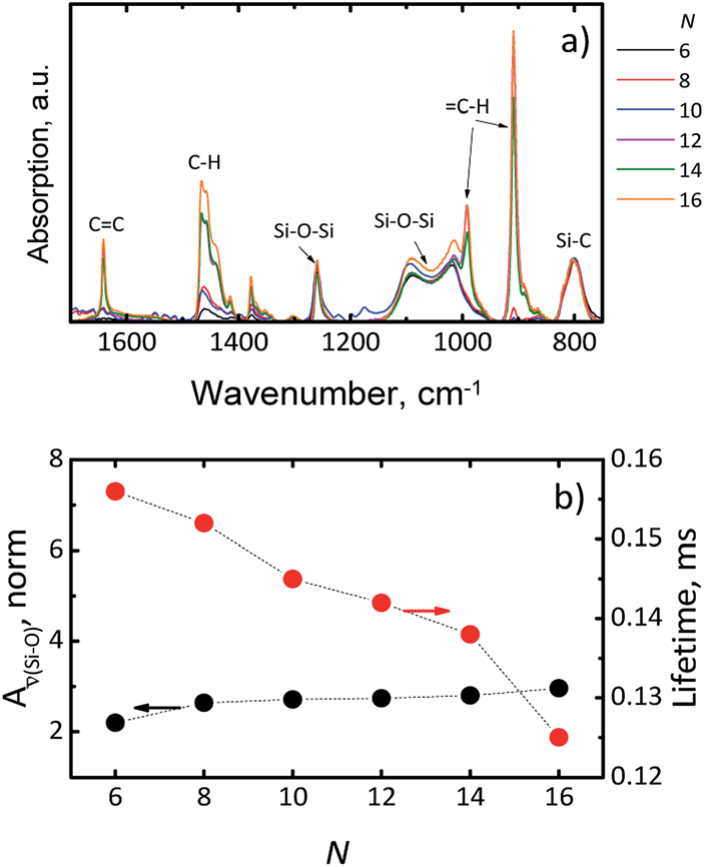
(a) Attenuated total reflectance infrared (ATR-FTIR) spectrum of Si-NCs with different capping ligands. *N* is the number of carbon atoms in the linear aliphatic chain of the capping ligand. (b) *A*_*ν̄*(Si−O)_ and luminescence lifetime as function of number (*N*) of carbon atoms in the capping ligand.

The lowest PLQY of 18 ± 2% was obtained for hexadecene-1. Our first hypothesis was that the partial surface oxidation of the Si-NCs is responsible for decreasing the PLQY in the case of ligands with longer carbon chains. This hypothesis might be supported by an analysis of FTIR spectra of capped Si-NCs. Fig. 5a displays a selected region of the FTIR spectra measured for Si-NCs capped with different ligands. We attribute the peak at 800 cm^−1^ to Si–C_(stretching)_ vibration, whereas the broad peak at 960–1140 cm^−1^ to Si–O_(stretching)_ vibration. The Si–C_(stretching)_ vibration can overlap with Si–O_(bending)_ peak (observed at 797–810 cm^−1^). However the Si–O_(bending)_ peak has typically much low intensity then Si–C_(stretching)_ and, thus, has minor impact in the further calculations. We did not observed the characteristic peak of Si–H vibration at 900 cm^−1^ that indicates about complete substitution of Si–H bonds due to oxidation and hydrosilylation. We expect that the ratio in the eqn (2) can provide qualitative information about surface oxidation of Si-NCs. We normalized the results in the way, that the integral *A*_*ν̄*(Si−C)_ = 1, so the value of the integral *A*_*ν̄*(Si−O)_ reflects relative amount of oxidation occurring at the Si-NCs surface. The large *A*_*ν̄*(Si−O)_ is the stronger oxidation occurs at the surface.2
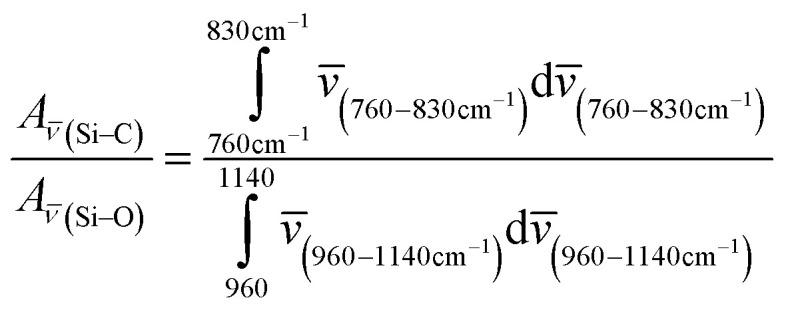


Fig. 5b indicates only the small increase of *A*_*ν̄*(Si−O)_ with the increase of the length of hydrocarbon chain. Thus, the surface oxidation is apparently not the key phenomenon responsible for the PLQY decrease. Surprisingly, washing several times the Si-NCs with hexane doesn't eliminates dodecene-1, tetradecene-1 and hexadecene-1 from Si-NCs. FTIR spectrums of Si-NCs capped with the long chain ligands display additional peaks corresponding to the –C=C– and =C–H bonds. The FTIR spectrums (Fig. 5a) also display a band at 1259 cm^−1^ that can be attributed to longitudinal phonons relating to Si–O–Si bond with the deformed bond angle ∼142°.^27^ This peak is well separated from the other Si–O–Si peak at 960–1140 cm^−1^ and its intensity does not change for different capping ligands. Though the existence such preferential configuration of Si–O–Si bond is unclear, we assume that this peak represents oxidation sites of Si-NCs surface originating from nature of SiO_*x*_ material because the similar peak is not observed in Si-NCs prepared from silsesquioxanes.^18,26,28,29^

An alternative explanation for the changes in PLQY with length of the capping ligands can be proposed using work of Aharoni *et al.*^30^ They described long-range electronic-to-vibrational energy transfer from excited quantum dots to matrix vibrational overtones. Fig. S4† depicts emission spectrums of Si-NCs capped with hexene-1 and hexadecene-1 and absorption spectrums of solvents (hexene-1 and hexadecene-1). The absorption spectrums of the alkenes (similar for all investigated alkenes) demonstrate C–H vibrational overtones with the absorption maximums peaking at 923–930 nm that perfectly match emission spectrums of Si-NCs. The matching is better for Si-NCs with long capping ligands. Thus, both solvent and capping ligands (forming the polymer corona) can act as an efficient quencher of excited Si-NCs. Indeed, Fig. 5b displays a decrease of the radiative lifetime in range *N* = 6 → *N* = 16 from 0.156 ms to 0.125 ms (luminescence decays are presented in Fig. S5†). The decrease of the lifetime signalizes about increase of non-radiative deactivation and is in good agreement with observed decrease of PLQY.

The presence of oxidized surface sites related to Si–O–Si bonds (from FTIR data) can also reduce PLQY of Si-NCs. We observed oxidation independently from used alkenes and methods of deoxygenation to prevent the oxidation. Apparently, these type of surface defects limits the highest achievable PLQY to 39% – or an average value of 34 ± 5% calculated from six independent syntheses – achievable by the method used in the current work.

**Fig. 6:**
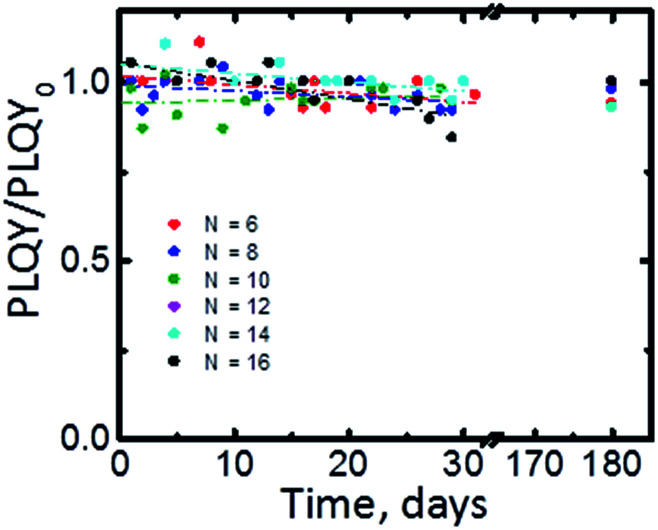
PLQY of Si-NCs stored in ambient as function shelf-life time for Si-NCs with different capping ligands. *N* is the number of carbon atoms in the linear aliphatic chain of the capping ligand.

To complete the characterization of the Si-NCs we systematically investigated the shelf-life of the synthesized materials. Fig. 6 displays PLQY values taken during the period six months. We did not observe significant altering of photoluminescence emission after six months of the storage in dark ambient conditions (PLQY/PLQY_0_ > 0.9). The part of the samples stored in a glovebox displays PLQY comparable with PLQY of samples stored under ambient conditions. FTIR spectrums of samples stored in a glovebox indicate also unchanged ratio between Si–C and Si–O bonds. These experimental observations confirm high stability of Si-NCs functionalized *via* microwave-assisted hydrosilylation.

## Experimental

### Materials

Silicon monoxide (99.9%, 325 mesh) was purchased from Sigma-Aldrich, hydrofluoric acid (48%) was purchased from Fisher Scientific, ethanol absolute, methanol HPLC grade, toluene (99%), dodecene-1 (94%), tetradecene-1 (96%), hexadecene-1 (98%), and octadecene-1 (96%) were purchased from Merck, hexene-1 (99%) and octane-1 (99%) were purchased from Acros, decene-1 (96%) and *n*-hexane spectroscopic grade were purchased from Alfa-Aesar.

### Synthetic procedures

#### Synthesis of hydrogen-terminated Si-NCs

1.0 g SiO_*n*_ was transferred to a quartz boat and reduced thermally under flowing H_2_ (5%) in Ar (95%) at 900 °C for 60 minutes in a quartz tube furnace. After annealing, the product was cooled down to room temperature and grounded using agate mortar. The fine powder was transferred to 100 ml Teflon vessel and 20 ml of absolute ethanol and 20 ml of HF acid solution 48% v/v were added and stirred magnetically for 150 minutes to remove the SiO_*x*_ matrix. The brown dispersion was transferred to a Teflon separatory funnel and extracted with 20 ml of corresponding alkene. The yellowish Si-NCs dispersion was discarded and transferred a microwave glass tube. Argon gas was purged into the dispersion for 20 minutes before reaction in a microwave (MW) reactor.

#### Hydrosilylation using MW reactor

The G30 tube was placed into MW chamber (Anton Paar Monowave 400) and heated thermally at 250 °C for 20 minutes in 600 rpm stirring. The temperature of the solution was monitored a ruby thermometer dipped inside the solution. After reaction complete, product was transferred to centrifuge tube and centrifuged at 4500 rpm for 10 min to separate large particles.

### Characterization

A UV-vis-NIR spectrophotometer (Perkin Elmer Lambda 950) was used to measure optical absorption of materials. A spectrofluorometer (Varian Cary Eclipse) was used for measuring excitation spectra. A FT-IR spectrometer (Bruker Vertex 70 with platinum ATR module) was used for measuring IR absorption. Dynamic light scattering (Anton Paar Litesizer 500) was used for measuring the size of particles dispersed in a liquid. PL emission spectra were measured with calibrated CCS200 spectrometer (Thorlabs) using 1 mW LED with *λ* = 375 nm for excitation. Absolute PLQY was measured using de Mello methods.^31^ The LED beam was focused by a lens and directed into an integrating sphere (Labsphere) with a diameter of 15 cm. An optical fiber with a diameter of 1 mm (FP1000URT, Thorlabs) was used for collection of the emission from the integrating sphere and transferring this to the spectrometer (CCS200, Thorlabs). During the absorption measurement (measurement of the LED at the direct and indirect excitation of the sample and empty sphere), short integration times, usually 20–50 times shorter than for UC detection, were utilized. All raw detected spectra were recalculated to give power spectra using an integration time value. The linearity of the signal *versus* integration time of CCD was proven experimentally. The spectral response of the whole detection system was calibrated using a calibration lamp (HL-3plus-INT-CAL, Ocean Optics) and the correction was further applied to the power spectra. For the PL lifetime measurements, time-correlated single photon counting (TCSPC) and a multichannel scaling (MCS) card (Timeharp 260, PicoQuant) were used. The modulation of the diode laser (Thorlabs 405 nm) was performed *via* a built-in function generator in the laser diode driver. In order to detect rise and fall times of the UC emission, the TTL signal from the laser diode controller was delayed by the use of a delay generator (DG645, Stanford Research Systems). The spectral separation of the photoluminescence was achieved *via* a double monochromator (DTMS300, Bentham) and the emission at specific wavelength was detected *via* a photomultiplier tune (R928P, Hamamatsu), mounted in temperature-cooled housing (CoolOne, Horiba).

TEM investigations were carried out on a TITAN 60-300 transmission electron microscope at accelerating voltage 300 kV.

## Conclusions

To conclude, MWH in very short step of 20 minutes allows getting Si-NCs with high PLQY of 39% and emission maximum of 860 nm. The highest PLQY is achieved through functionalisation with hexene-1 – the shortest ligand among investigated alkenes. We attribute increase of PLQY in case of capping with hexene-1 with reduce of long-range electronic-to-vibrational energy transfer from quantum dots to matrix vibrational overtones. Furthermore, Si-NCs prepared from silicon monoxide SiO_*n*_ bear sufficient number of surface defects associated with deformed Si–O–Si bonds, which can intrinsically limit the values of PLQY prepared form this type of precursor. Meanwhile, MWH results highly luminescent Si-NCs with long shelf-life at the ambient conditions.

## Conflicts of interest

There are no conflicts to declare.

## Supplementary Material

RA-008-C7RA13577G-s001
