# Supplementary material for: Highly photoluminescent and stable silicon nanocrystals functionalized via microwave-assisted hydrosilylation
Source: RSC Adv. 2018 Mar 12;8(18):9979–84. doi: 10.1039/c7ra13577g (PMC9078736; doi:10.1039/c7ra13577g)
Supplement: RA-008-C7RA13577G-s001 [file RA-008-C7RA13577G-s001.pdf]

Electronic Supplementary Material (ESI) for RSC Advances.
This journal is © The Royal Society of Chemistry 2018

Supporting information:- Highly Photoluminescent and Stable Silicon Nanocrystals Functionalized via Microwave-Assisted Hydrosilylation

Deski Beri[a], Dmitry Busko[a], Andrey Mazilkin[b], Ian A. Howard[a,c], Bryce S. Richards[a,c] and Andrey Turshatov[a]

a. Institute of Microstructure Technology, Karlsruhe Institute of Technology, Hermann-von-Helmholtz-Platz 1, 76344, Eggenstein-Leopoldshafen, Germany.
b. Institute of Nanotechnology, Karlsruhe Institute of Technology, Hermann-von-Helmholtz-Platz 1, 76344 Eggenstein-Leopoldshafen, Germany.
c. Light Technology Institute, Karlsruhe Institute of Technology, Engesserstrasse 13, 76131 Karlsruhe, Germany.

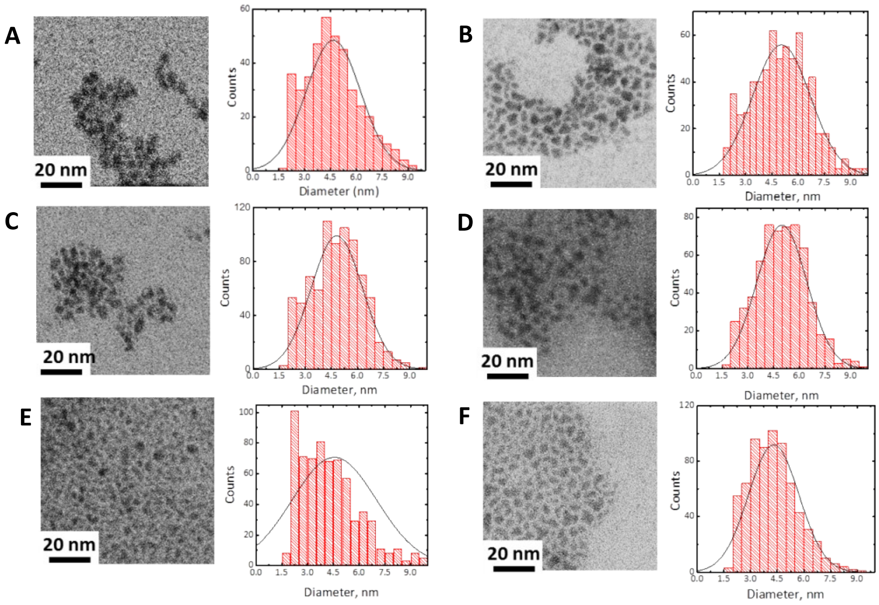

Fig. S1 TEM images and particle distribution of of free standing Si-NCs capped with hexene-1 (A), octane-1 (B), decene-1 (C), dodecene-1 (D), teteradecene-1 (E) and hexadecane-1 (F).

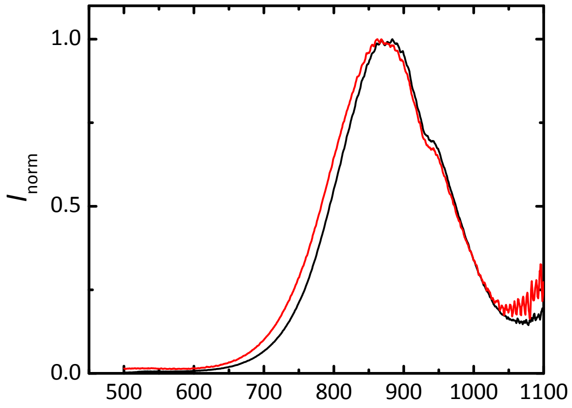

Fig. S2 Normalized luminescence spectrums of Si-NCs prepared through MW heating (20 min, 250 °C) – black line and conventional heating (18 hours, 175 °C) – red line. Capping ligand is decene-1. PLQY in case of MW heating is 27 %. PLQY after conventional heating is ..%

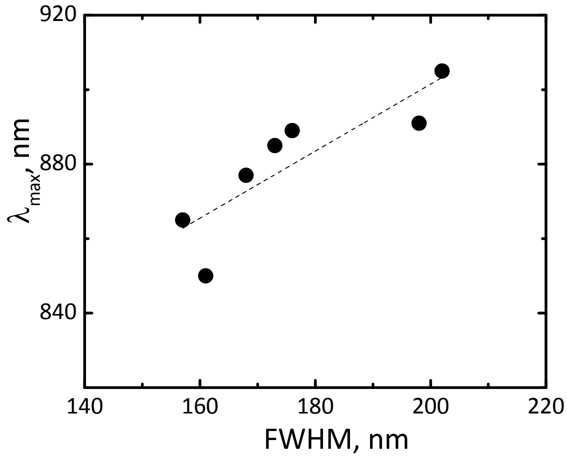

Fig. S3. Position of luminescence maximum as function of the full width at half maximum (FWHM) derived for the Si-NCs luminescence peaks (Fig. 2). Fig. S3 displays red shift in the position of luminescence maximum observed for Si-NCs with broad size distribution (e.g. broad luminescence peak)

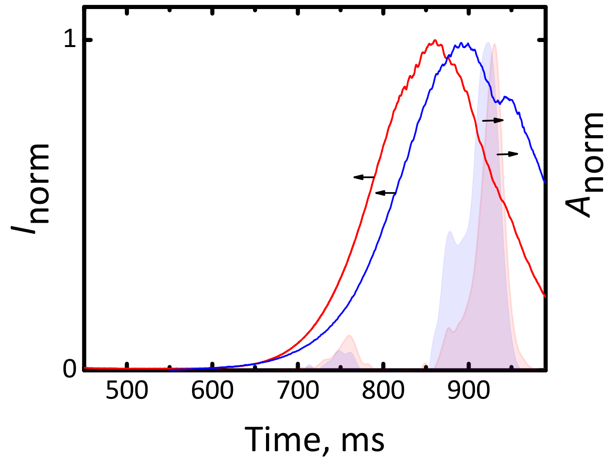

Fig. S4. Emission (I_{norm}) of Si-NCs capped with hexene-1 (red) and hexadecene-1 (blue) dispersed in hexene-1 and hexadecene-1, correspondingly. NIR absorption (A_{norm}) of hexene-1 (red) and hexadecene-1 (blue).

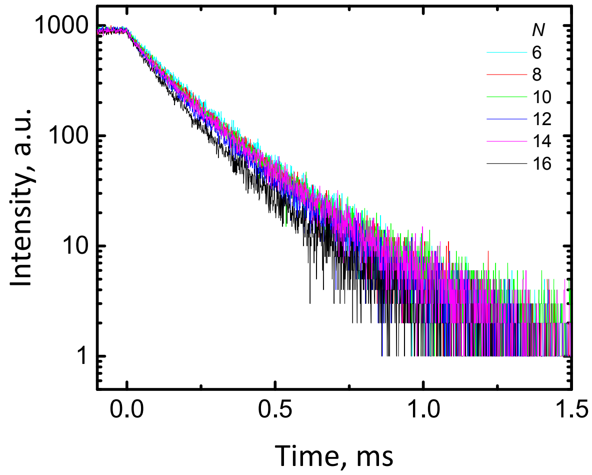

Fig. S5 Luminescence decays of Si-NCs with different capping ligands. *N* is the number of carbon atoms in the linear aliphatic chain of the ligand.
